# Assessment of acetochlor use areas in the sahel region of Western Africa using geospatial methods

**DOI:** 10.1371/journal.pone.0230990

**Published:** 2020-05-01

**Authors:** Cornelis Hoogeweg, Brian Kearns, Naresh Pai, Mark Thomas, Ian van Wesenbeeck, Annette Kirk, Jim Baxter, on behalf of the Acetochlor Registration Partnership

**Affiliations:** 1 Waterborne Environmental, Inc, Leesburg, Virginia, United States of America; 2 Bayer U.S.—Crop Science Division, Chesterfield, Missouri, United States of America; 3 Corteva Agriscience, Indianapolis, Indiana, United States of America; United Nations University Institute for Natural Resources in Africa, GHANA

## Abstract

The goal of this study was to determine the co-occurrence between acetochlor use on crops and potentially vulnerable soils in the Permanent Interstate Committee for Drought Control in the Sahel region of Western Africa. Acetochlor, a pre-emergence herbicide, is used primarily on row crops and has the potential to reach groundwater or surface water following a rain event shortly after application. Off-field transport is often determined by soil properties; therefore, soils within potential use areas were assessed and mapped to establish areas with soils vulnerable to leaching and/or runoff. Corn and cotton production areas were used as surrogate crops for high potential use areas of acetochlor within areas identified using GlobCover land use data and the Spatial Production Allocation Model agricultural statistics data. The geospatial analysis identified approximately 462 million ha of potentially vulnerable soils in the Sahel region of which 65.7 million ha are within agricultural areas. An adjustment for corn and cotton production areas showed that 2.2 million ha or 3.3% of agricultural fields could have potential restrictions for acetochlor use. Approximately 0.159 million ha of soils or 0.24% of agricultural fields are in the presence of shallow groundwater, defined by depth < 9 m. In addition, 0.0128 million ha or 0.02% were determined to be adjacent to surface water bodies. To understand the uncertainty associated with the use of specific land cover datasets, an overlay assessment was conducted using alternative data sources. Overlap between selected land cover datasets in the Sahel region varies and ranges from 24.7% to 75.5% based on a merged 2009 GlobCover and CCI LC datasets. In comparison with the merged 2005 and 2009 GlobCover dataset, the cropland overlaps range from 38.9% to 85.0%. This demonstrates that the choice of land cover dataset can have a significant impact on a spatial assessment. Results from this assessment demonstrate that only a small fraction of vulnerable agricultural soils across the region may be a risk for contamination by acetochlor of groundwater or surface resources, based on product label recommendations. Given the availability of spatial data in a region, the methods contained herein may additionally be used in other localities to provide similar information that can be helpful for water quality management.

## Introduction

Acetochlor (2-Chloro-N-(ethoxymethyl)-N-(2-ethyl-6-methylphenyl) acetamide) belongs to the group 15 “chloroacetamide” class of herbicides and is registered and approved for use in several crops and in more than forty countries around the world. Acetochlor was first registered in the United States in 1994 by the Acetochlor Registration Partnership (ARP). Acetochlor is commonly used on cotton, corn, soybean, sugar beet, and several other crops and provides control of annual grassy weeds and many annual broadleaf weeds. Acetochlor controls weeds by inhibiting growth of seedling shoots [1] and needs to be applied before weeds emerge to be effective. Therefore, it is typically applied just before or after planting of the crop.

Registration of the use of acetochlor in the United States prompted formation of the Acetochlor Registration Partnership (ARP), which seeks to “ensure the effective use and stewardship of products containing acetochlor” [2]. The ARP stewardship program contains several elements, including education and promotion of best management practices for surface water and groundwater protection. The ARP established a set of specific criteria that identify potentially vulnerable soils (i.e. coarse-textured permeable soils of low organic matter) for acetochlor [3]. The three potentially vulnerable soils of interest are: 1) sands with less than 3 percent organic matter; 2) loamy sands with less than 2 percent organic matter; 3) sandy loams with less than 1 percent organic matter. Hereafter, these vulnerable soils will be referred to as ARP 3-2-1 soils. For the protection of groundwater, a set-back of 15 m (50 ft) from wells is required on US product labels for applications on these vulnerable soil areas with shallow groundwater, meaning groundwater less than 9 m (30 ft) deep [4]. The current acetochlor label in the US does not require a buffer between the application area and fresh water sources; however, other restrictions, such as prohibiting applications to powdery-dry or light sandy soil under windy conditions, are included on the label, and separate Best Management Practices (BMPs), which encourage consideration of buffers, erodibility of land, irrigation, and amount of rainfall, are promoted, to avoid surface water contamination.

Applications made in vicinity of waterbodies could potentially result in off-site movement of acetochlor, particularly if applied before a significant rainfall, from the field to waterbodies. As a voluntary BMP, surface water protection relies on the implementation of a 20 m (66 ft) application area buffer from the edge of rivers, creeks, streams and ponds [2]. Buffer distance may vary; for example, the state of Minnesota in United States now requires perennial vegetation buffers of up to 15.2 m (50 ft) for all pesticides used along lakes, rivers, and streams and buffers of 5 m (16 ft) along ditches [5].

As part of product stewardship, the ARP created a comprehensive soil map for the continental US [2] showing areas with restricted soils based on the above listed criteria. The map details with high resolution where acetochlor use is allowed and provides an overview of which groundwater resources may be vulnerable to acetochlor in the US. A similar map has not yet been created for Africa, in part because the data sources and geospatial methods have become available only recently. In regions like the Sahel, concerns may exist that, following application, acetochlor might contaminate water resources depending on the temporal proximity of application to vulnerable ARP 3-2-1 soils. Whether water runs off or percolates through the soil is largely dependent on the characteristics of the soil itself [6]; soil with poor infiltration capacity might cause water to run off into surface waters, while soil types with high infiltration capacity, for example sandy soils, allow water to percolate into groundwater sources. It is the case, however, that multiple factors must co-occur to indicate potential vulnerability of a water resource [6].

Historically, the Western Africa Sahel area has frequently been affected by drought, most notably the drought of the early 1970s which resulted in near total loss of all agricultural crops and up to 70 percent loss of cattle. To address and mitigate drought concerns, the CILSS (French: Comité permanent inter-État de lutte contre la sécheresse au Sahel) was formed as a consortium to invest in the search for food security and in the fight against the effects of drought and desertification for a new ecological balance in the Sahel. In addition, members of the CILSS work towards the standardization of regulations relating to seeds and pesticides [7].

To assess the potential environmental risk of acetochlor, the ARP developed a comprehensive soil map to highlight areas in the West Africa CILSS region with potentially vulnerable soils. The goal of this study was to determine the proportion of areas in the CILSS region where acetochlor usage might present a risk for groundwater or surface water contamination based on salient environmental factors. The results from this study, including the vulnerable soil map, could be a useful and novel data resource to both acetochlor applicators as well as regulators, to inform the development of best management practices for the sustainable use of acetochlor in the CILSS region.

## Materials and methods

### Study region

**Fig 1:**
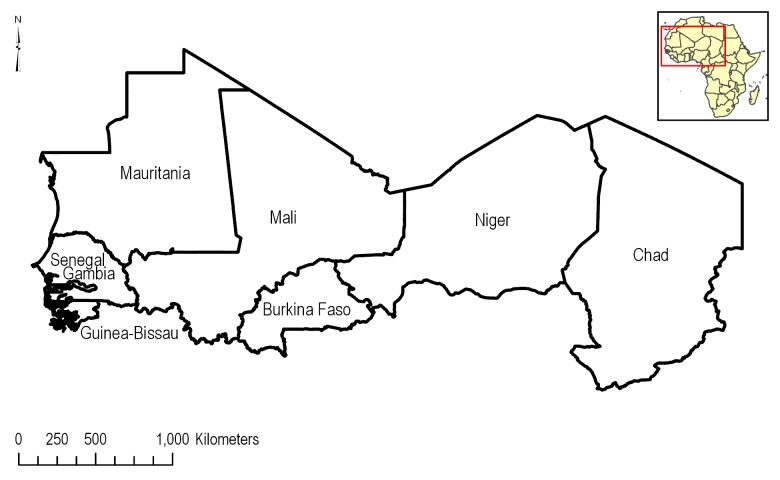
CILSS countries considered in the assessment of vulnerable soils under corn and cotton production.

The study area (5,260,981 km^2^) considered in this assessment encompassed several of the members of the CILSS region of West Africa. This region consists of the countries Burkina Faso (273,981 km^2^, 5.2% of total study area), Cape Verde (Republic of Cabo Verde; 4,091 km^2^, 0.1%), Chad (1,270,749 km^2^, 24.2%), Gambia (10,626 km^2^, 0.2%), Guinea-Bissau (33,741 km^2^, 0.6%), Mali (1,252,498 km^2^, 23.8%), Mauritania (1,038,902 km^2^, 19.7%), Niger (1,181,106 km^2^, 22.5%), and Senegal (195,197 km^2^, 3.7%) (Fig 1). At the time of this study, Benin, Ivory Coast, and Togo were not yet official members, thus were not included in this assessment. CILSS countries are part of the Sahel, which is a transitional zone in Africa between Sudanian Savanna and rainforests in the south and the Sahara Desert in the north. The area stretches from the Red Sea in the East to the Atlantic Ocean in the west. Average rainfall is between 0.20 and 0.60 m, with precipitation occurring mainly from May through September. Most rainfall occurs in the southernmost portion of the region.

### Data sources and processing

Spatial agricultural data, derived from global and continental datasets, were analyzed in a geographic information system (GIS) software to determine potential acetochlor use areas, based on crops grown. Following the establishment of the potential use area (PUA), soil characteristics in agricultural areas were described and compared against the ARP soil criteria within the PUA. Soils that were classified as vulnerable under the ARP 3-2-1 criteria were overlaid with groundwater and surface water data as well as agricultural use sites to determine areas where agricultural practices potentially benefitting from the application of acetochlor may present a threat from runoff or leaching.

#### Land use and land cover

**Fig 2:**
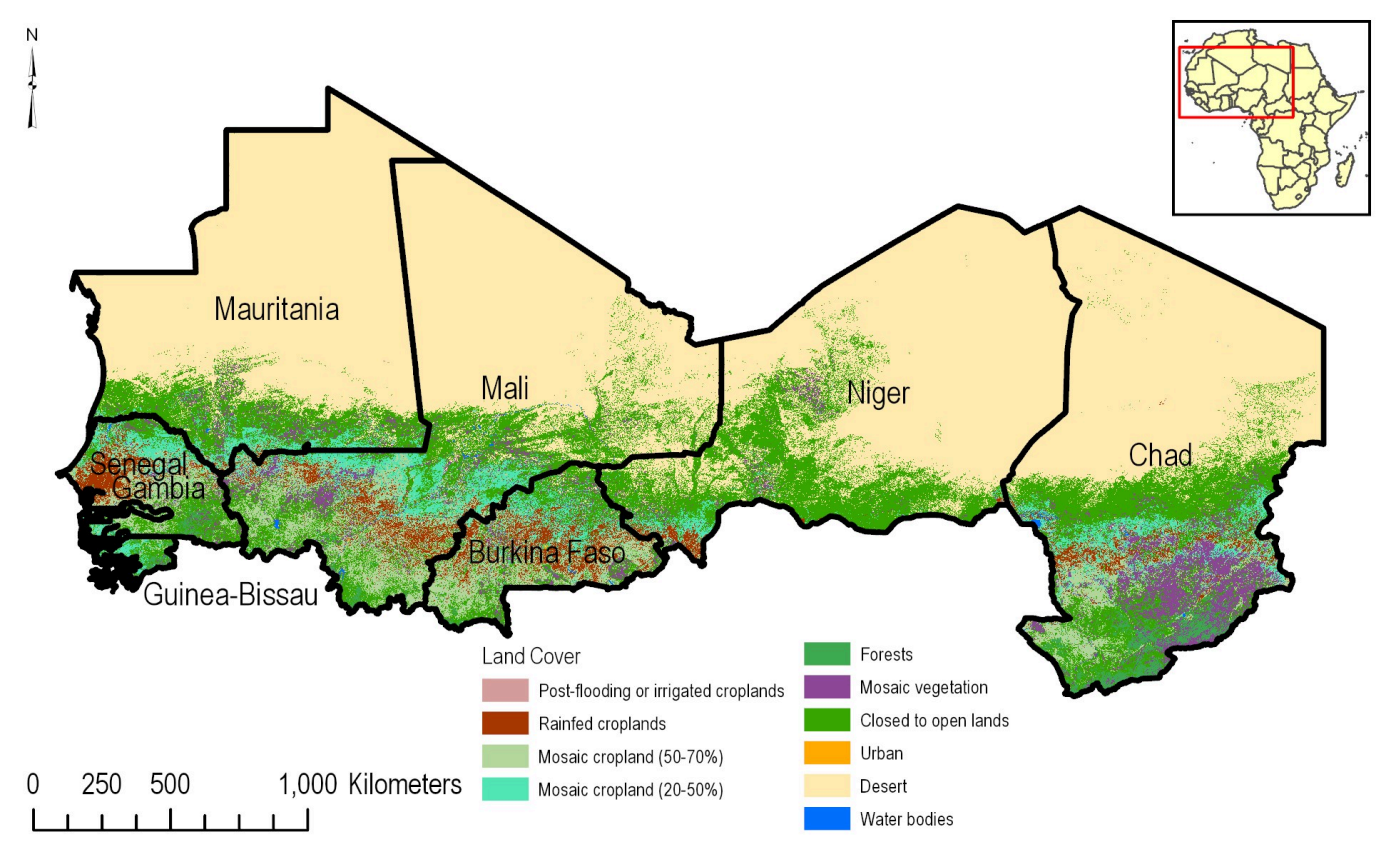
European Space Agency GlobCover land use [8] depicting the main land use classes in the western Africa CILSS countries.

Agricultural land use in the CILSS region was determined using the 2009 European Space Agency (ESA) GlobCover dataset [8]. GlobCover 2009 is a 300-m resolution dataset based on Medium Resolution Imaging Spectrometer (MERIS) full-resolution satellite imagery for the period January 1 to December 31, 2009 with each pixel roughly representing 9 ha. GlobCover 2009 has an overall accuracy of 67.5%, with highest accuracies occurring in Europe. For the CILSS region, 101–200 temporal images were used for each 5-by-5-degree area covered in a single shot by the MERIS satellite [8]. The resulting product, GlobCover 2009, has 22 different land use classes covering agriculture, forest, urban areas and waterbodies (Fig 2).

#### Agricultural statistics

**Fig 3:**
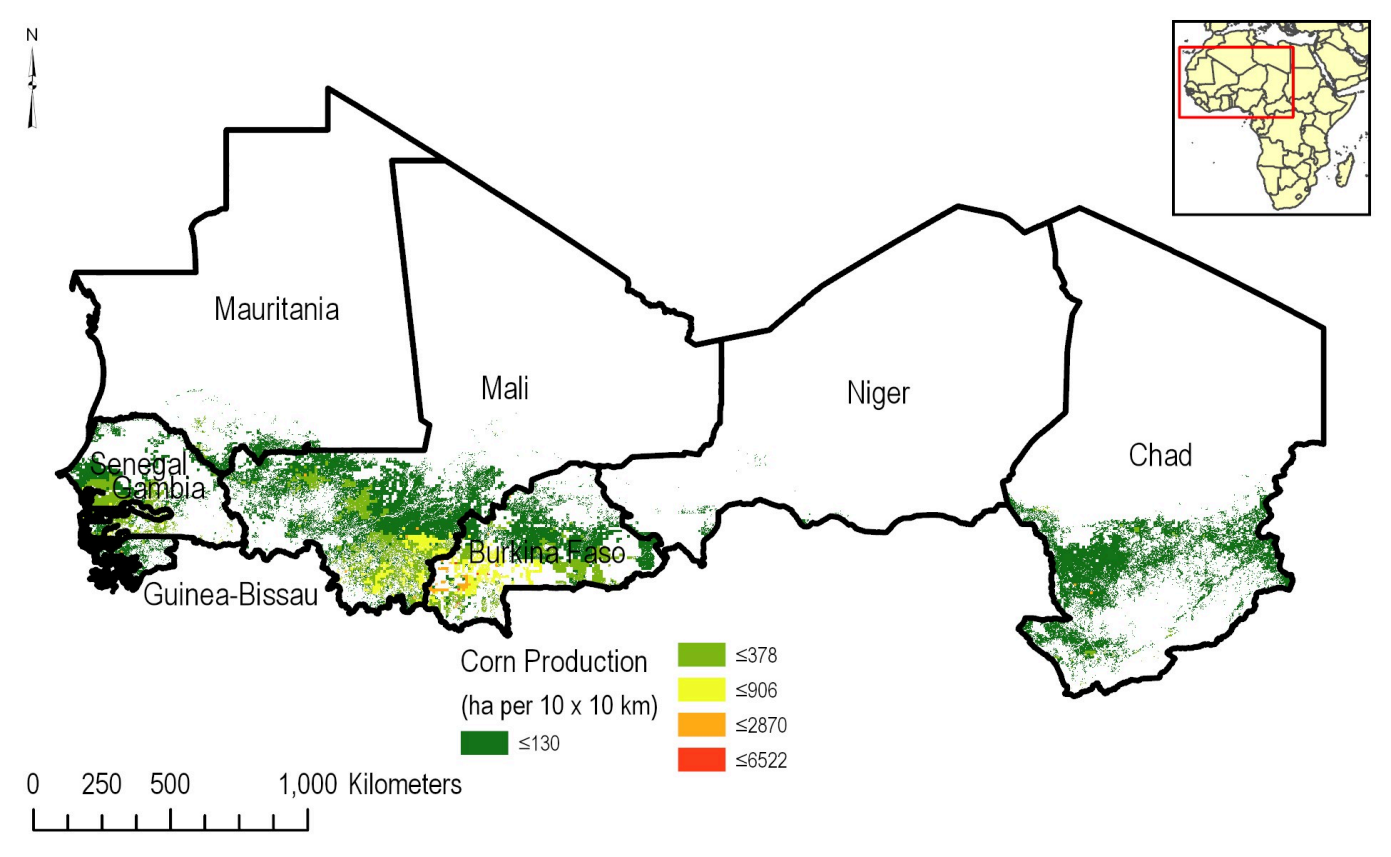
Distribution of corn production in the CILSS based on the 10 km x 10 km mapSPAM [9] data.

**Fig 4:**
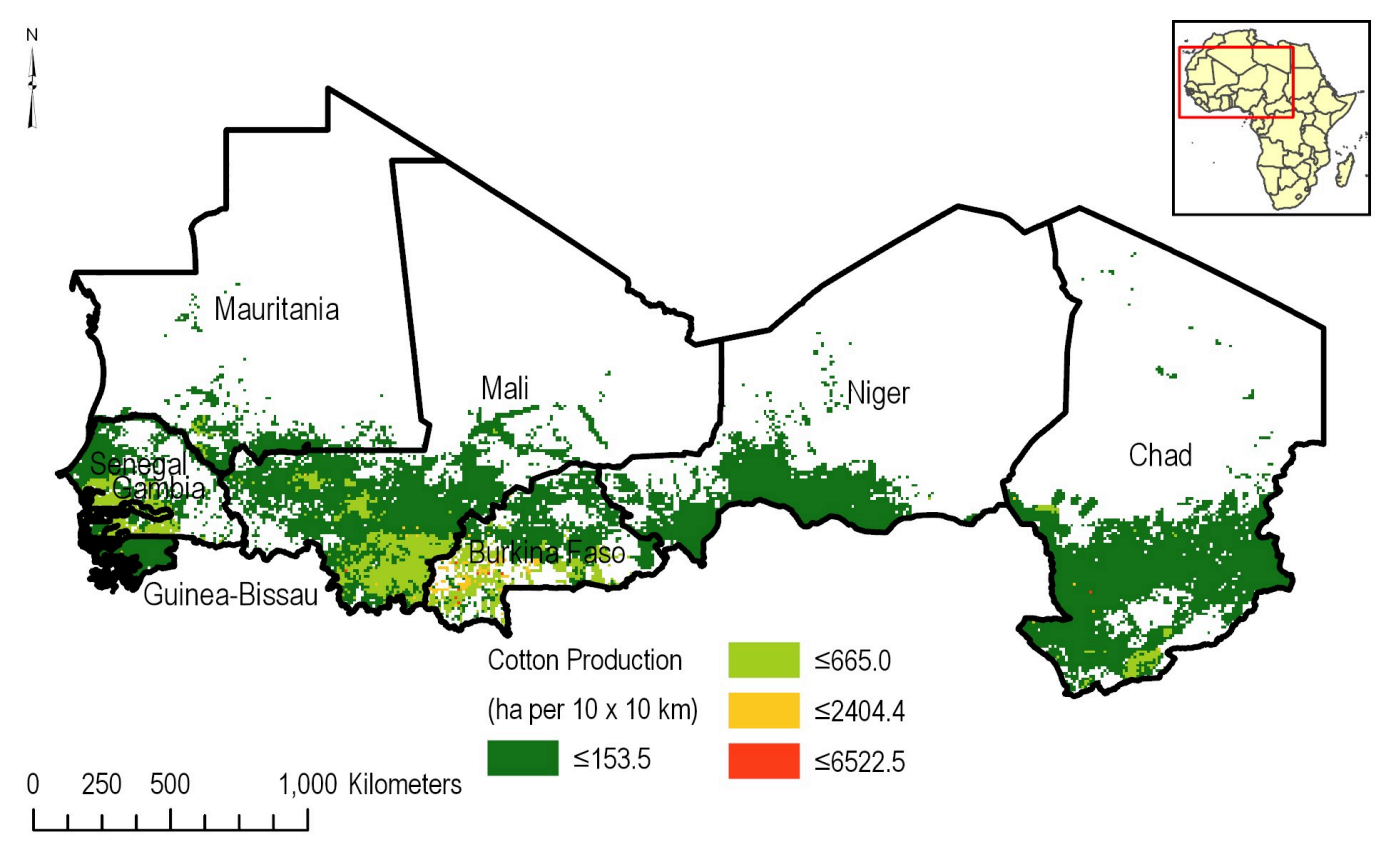
Distribution of cotton production in the CILSS based on the 10 km x 10 km mapSPAM [9] data.

An understanding of the spatial distribution of corn (Fig 3) and cotton (Fig 4) across the CILSS region is paramount to this assessment. The Spatial Production Allocation Model (SPAM) was used to gain insights into cropping patterns [9]. MapSPAM is used by the International Food Policy Research Institute (IFPRI) as part of the organization’s global change research programs, such as the Harvest Choice, as well as regional research and development priority setting within IFPRI for West Africa. Information from this dataset includes harvest area, physical area, production and yield, and is available for 68 crops. Specialized crop datasets are available for irrigated, rainfed and total crop area. MapSPAM provides all crop information at a 10 km x 10 km grid level.

#### Soil data

**Fig 5:**
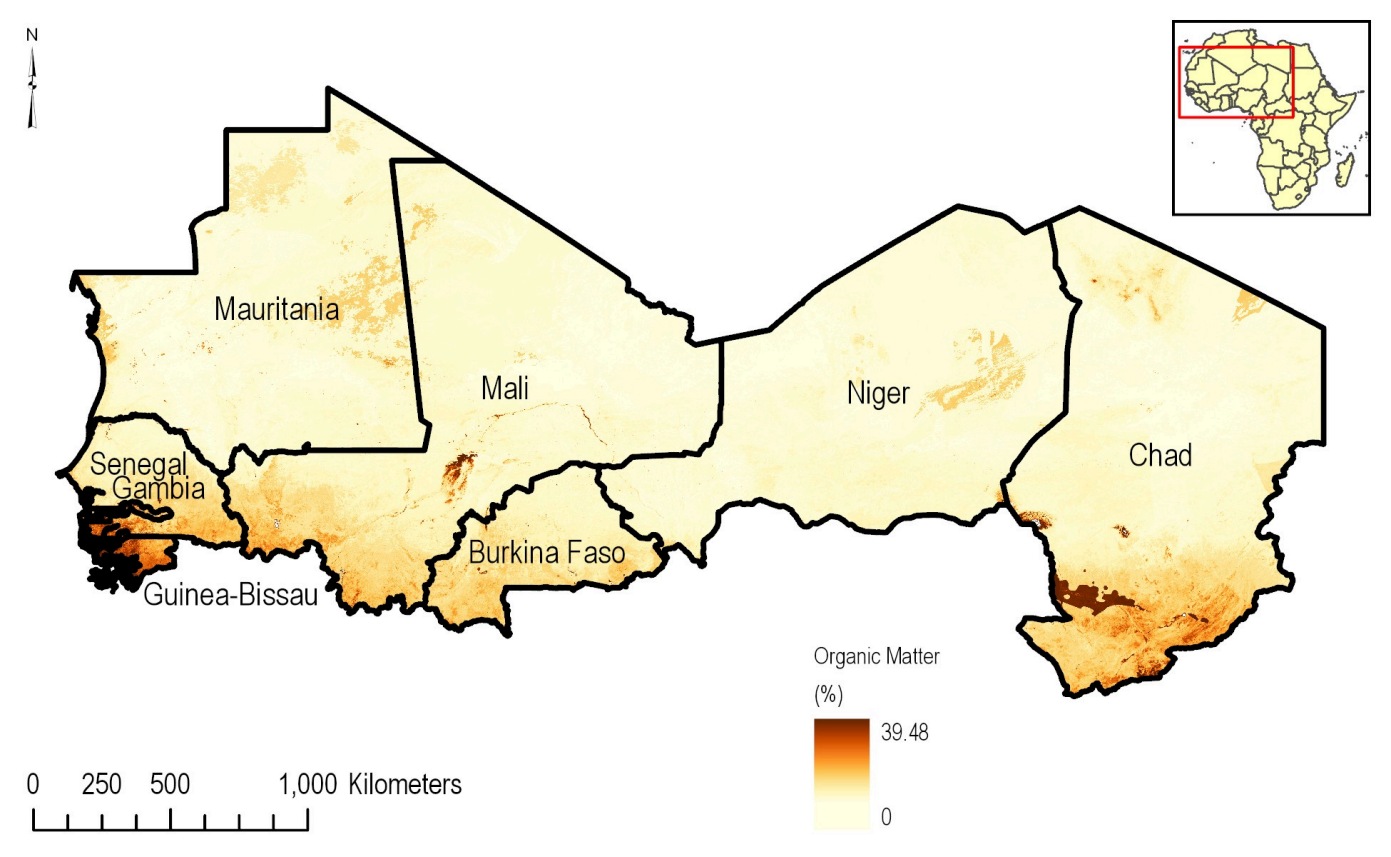
Distribution of topsoil (0–15 cm depth) organic carbon content in CILSS based on the SoilGrid [10] data.

Soil data for this assessment was sourced from the SoilGrid dataset [10]. The 2017 release of SoilGrid is derived from a complex non-linear machine-learning model in combination with remote-sensing-based soil covariates. These covariates were derived from Moderate Resolution Imaging Spectroradiometer (MODIS). land products, Shuttle Radar Topography Mission (SRTM), Digital Elevation Model (DEM) derivatives, climatic images, and global landform and lithology maps and used to predict soil properties across the globe based on over 150,000 soil profiles globally. Resulting from this effort were soil profiles that were standardized at seven depths (0, 5, 15, 30, 60, 100 and 200 cm) for organic carbon (Fig 5), soil texture, particle size distribution (sand, silt, and clay), pH, bulk density, and more. The final dataset has a resolution of 250 m and contains over 280 raster layers representing 7 different depths describing the soil taxonomy and soil physical properties.

#### Surface hydrography

**Fig 6:**
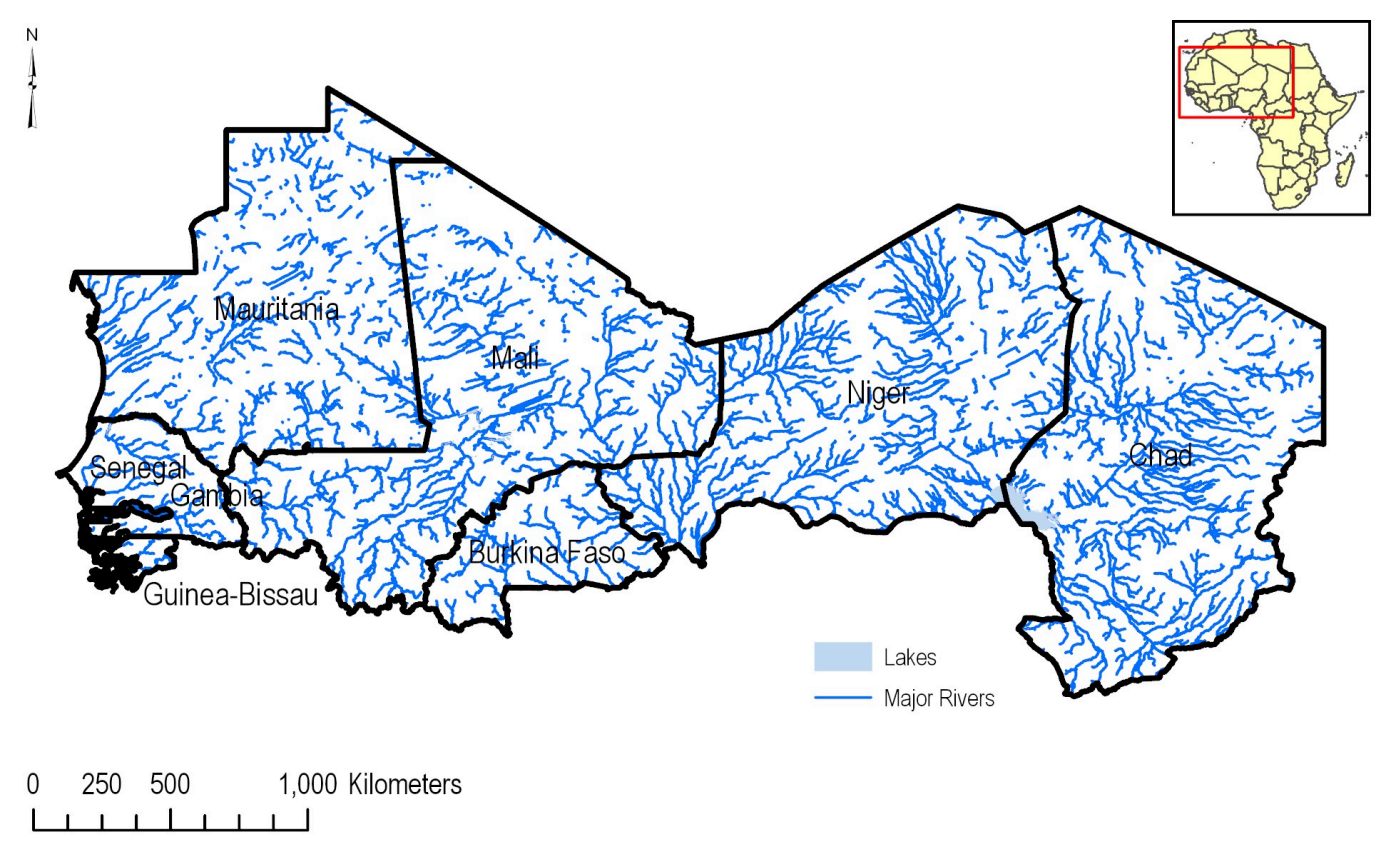
Location of stream flow paths in the CILSS region developed from the HydroSHEDS data. Only rivers with number of upstream cells greater than 5000 are shown.

Surface hydrography data was used in this analysis to determine which agricultural areas were adjacent or in close proximity to both static and flowing surface water bodies (Fig 6). A river network dataset with line geometry was obtained from the HydroSHEDS database [11]. HydroSHEDS is a global database that delivers several important hydrological metrics, including: rivers (as lines), larger scale watersheds, void filled elevation, hydrologically conditioned digital elevation models, drainage direction, and flow accumulation. Data for these metrics are remotely sensed from the Shuttle Radar Topography Mission (SRTM), and are available at 3 arc-second, or approximately 300 m, resolution. For surface water bodies that needed to be represented as areas (e.g., lakes), ESRI’s World Hydro Base map [12] was used.

#### Groundwater table depth

**Fig 7:**
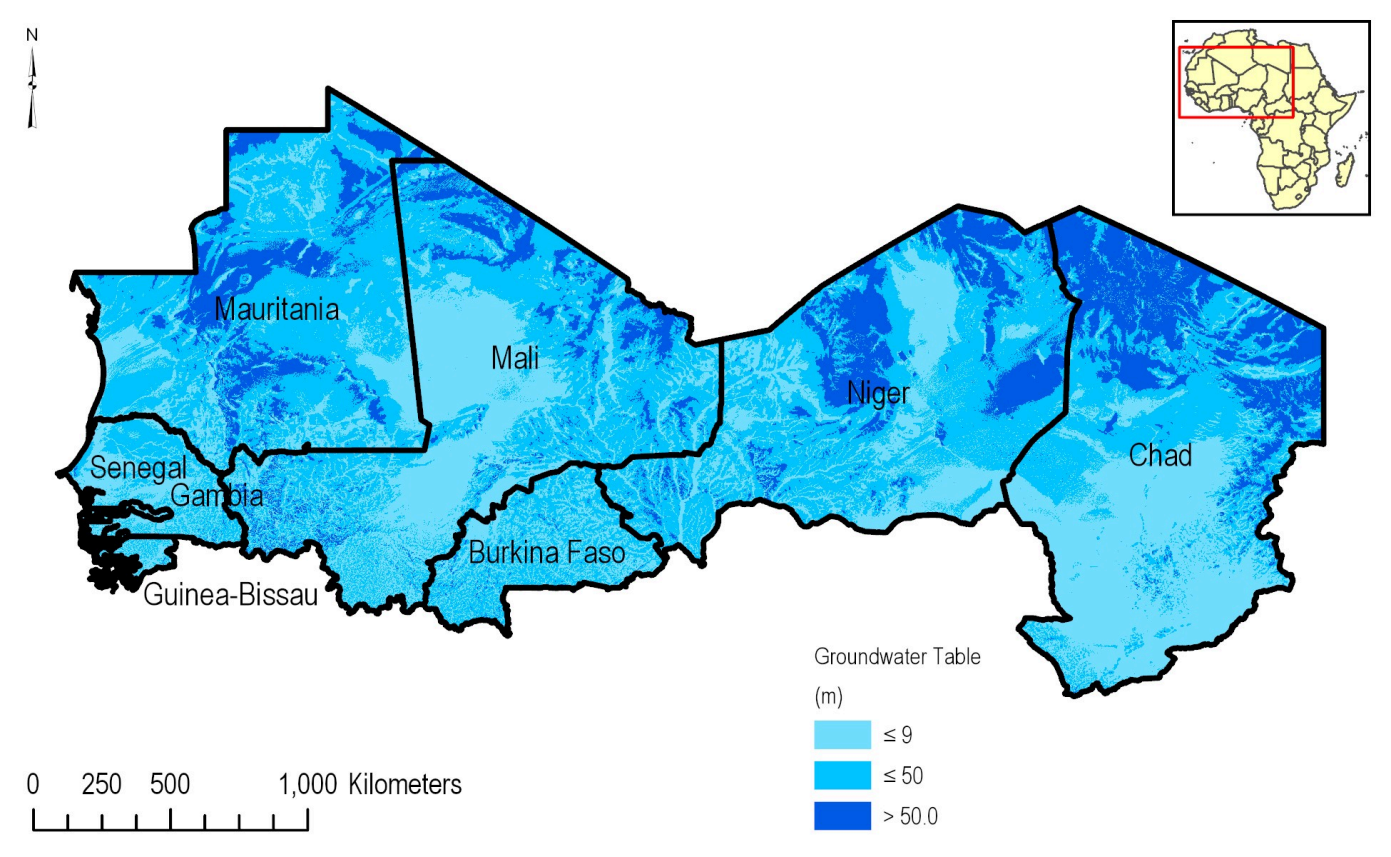
Spatial distribution of groundwater depth in the CILSS [13].

While governmental bodies provide large input datasets for areas such as North America and Western Europe, governmental data was largely unavailable for Western Africa. Depth to groundwater table for Western Africa and the CILSS region was obtained from Fan et al. [13] (See Fig 7). Fan et al.’s analysis, which produced continental-scale estimations of groundwater depth, relied on interpolating existing measurements for groundwater depth across vast areas, including 431 monitoring sites in Africa, many of which are in the CILSS. The dataset is driven by an existing groundwater model that relies mainly on modern climate, terrain, and sea level to drive predictions and evokes a hydrologic equilibrium ([13]–S1 Fig). This allowed for the determination of which groundwater areas were likely to be shallower than 9 m in depth.

### Geospatial analysis

**Fig 8:**
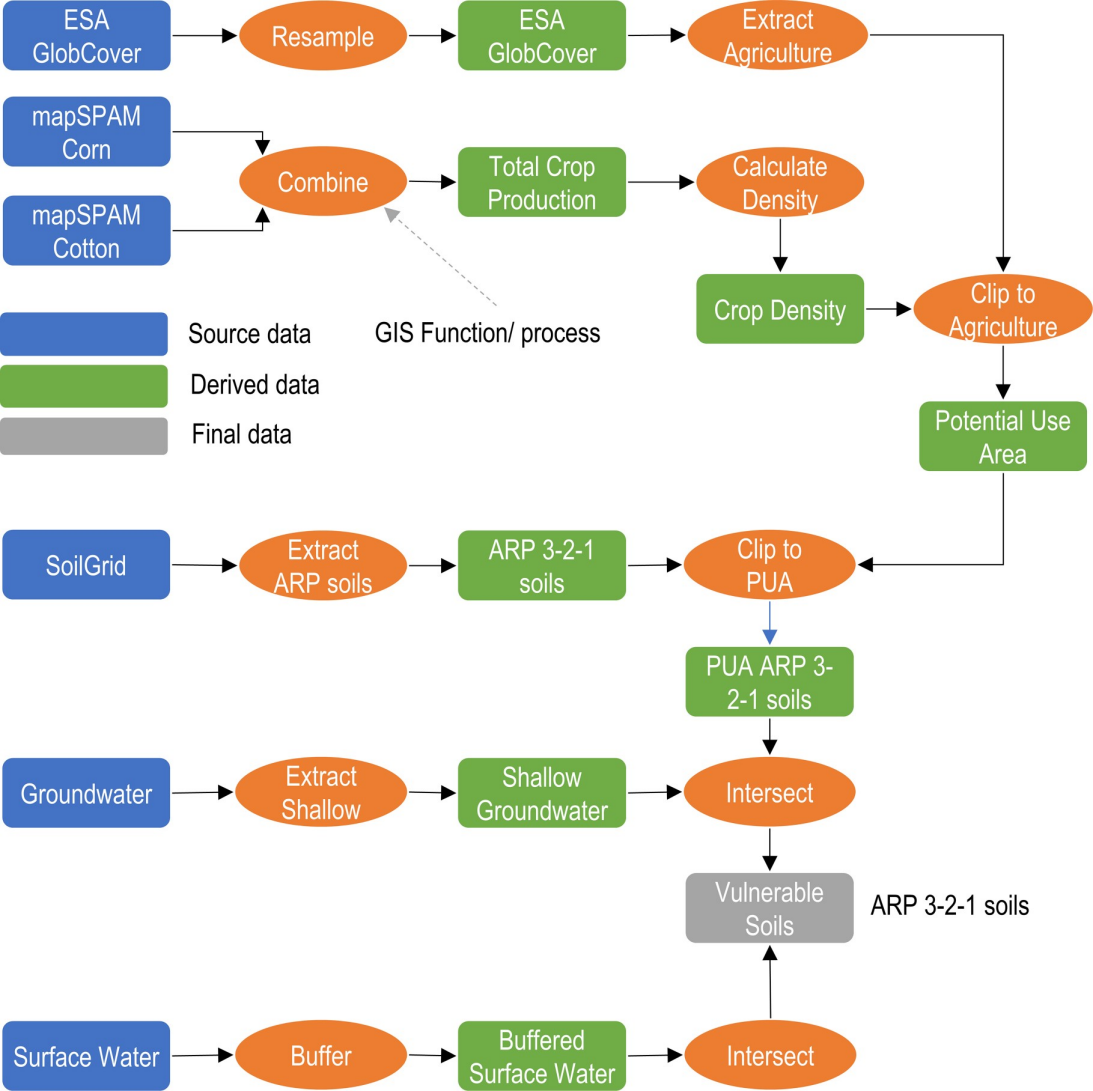
GIS data processing flow showing the source data, derived data, final datasets and key GIS processing steps.

All spatial analyses were conducted in ESRI ArcGIS 10.5 [14] software environment. Fig 8 shows the details of the GIS data processing flow. CILSS political boundaries were sourced from ESRI’s atlas dataset [12]. Once extracted, the country boundaries of the CILSS members functioned as an extraction mask for all other datasets in the analysis. Agricultural areas in the CILSS countries were defined as areas being members of the following four ESA GlobCover classes: 1) post-flooding or irrigated crops; 2) rain fed croplands; 3) mosaic cropland (50–70%); 4) vegetation (grassland/shrubland/forest) (50–70%)/ cropland (20–50%). These four classes were extracted and were used as a spatial filter in combination with the corn and cotton layers from MapSPAM to form the PUA data layer. Using ArcGIS Desktop, the resolution of the 300 m land cover layer was resampled to 250 m to match the soils data resolution using the nearest option.

Developing a combined dataset of production areas for corn and cotton was a multi-step process. First, the total production areas for corn and cotton were combined into a single dataset using the GIS’ “Combine” geoprocessing tool and adding a new field with the sum of the total production areas. This layer provided the overlap spatial extent for crop production in the CILSS region. Next, the crop density was calculated using the equation:

CrpD=CPa/PIXa (1)

Where

CrpD = crop density (-)

CPa = reported crop production (ha) per pixel

PIXa–pixel area (ha), which is 10,000 ha

Before the total area of corn and cotton overlapping vulnerable soils can be calculated, it is necessary to calculate the number produced within a single cropland pixel. The reported production density of 10 km x 10 km was rescaled to 250 m x 250 m grids. Within each 10 km x 10 km pixel, 1600 pixels were derived to represent the 250 m x 250 m grid. To determine the number of cropland pixels within each mapSPAM pixel, the raster layer was vectorized using ArcGIS Desktop Raster-to-Polyline geoprocessing tool. The Zonal Statistics Tool was then used to determine the number of cropland pixels presented within each mapSPAM pixel. With this reformatted map layer, the crop density adjusted to 250 m was calculated using the following equation:

aCrpd=CrpDx(1,600/nGCp) (2)

Where

aCrpD = Adjusted crop density (-)

CrpD = Crop density (-)

**Fig 9:**
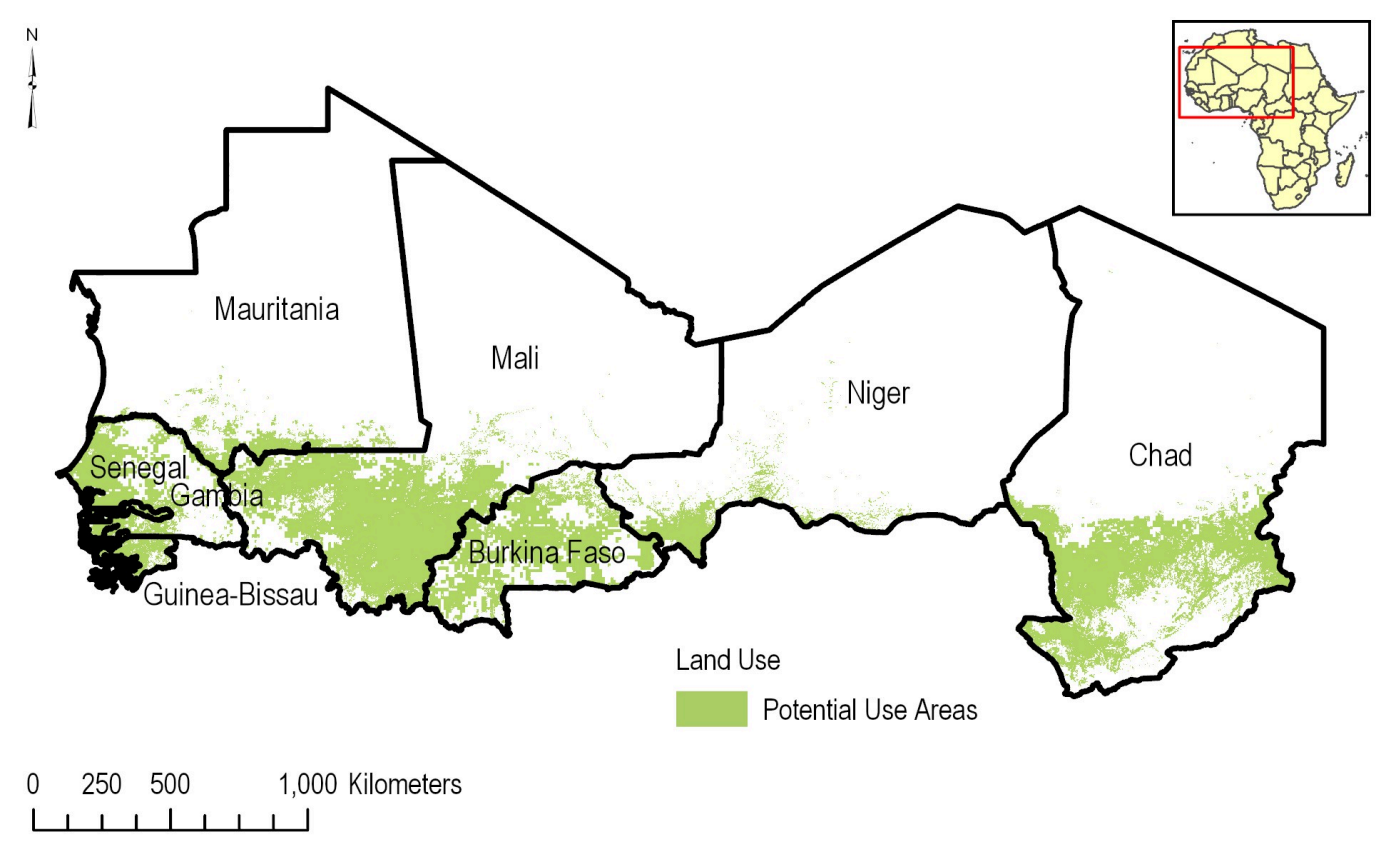
Potential Use Area (PUA) in the CILSS. The PUA consists of the combined corn and cotton production area clipped to the agricultural land uses.

nGCp = number of GlobCover pixel overlapping with a mapSPAM 10 km x 10 km pixelThe combined corn and cotton crop density layer was clipped using the GlobCover agricultural layer as an extraction mask to form the potential use area (PUA) layer (Fig 9).

Prior to determining which soils adhered to the ARP 3-2-1 soils criteria, the SoilGrid texture layer (which uses USDA texture classes) and the 0–5 cm and 5–15 cm organic carbon content layers were combined into a single dataset. A depth-weighted area organic matter (OM) layer was developed using a raster calculator to calculate the depth-weighted average 0-15cm organic carbon content. The resulting layer was multiplied by 1.724 to convert organic carbon into OM content using the ARCGIS raster calculator. The 0–15 cm layer was used to represent to top soil layer of the profile. Soils adhering to the ARP 3-2-1 soils criteria were extracted from the combined texture-organic matter dataset. Soils of interest included “sand” with < 3.0% OM, “loamy sand” with < 2.0% OM, and “sandy loam” with < 1.0% OM.

Using the HydroSHEDS river network and the World Hydro Base Layer, a highly conservative 200 m buffer zone was created for surface water bodies (rivers and lakes). The results from the 200 m buffer zone suggests that any analysis conducted at a closer distance, e.g. 20 m (66 ft) buffer to surface water, would have resulted in no vulnerable areas in proximity to surface water. A buffer distance of at least half the raster resolution (250 m) is required as the function operates on the raster cell center. This buffer dataset was used as a spatial filter to determine ARP soils in proximity or adjacent to surface water bodies.

All groundwater areas with table depth of less than 9 m (30 ft) were extracted to determine the locations of shallow groundwater. The resulting groundwater data layer was used to extract soils in agricultural areas and overlapping with shallow groundwater. The groundwater dataset was resampled to a resolution of 250 m to match the SoilGrid dataset spatial resolution. ARP 3-2-1 soil areas were combined with areas of shallow groundwater are characterized as “vulnerable soils” in the PUA.

The ARP 3-2-1 soils layer was combined with the surface water buffer layer to determine which crop areas on vulnerable soils were adjacent to surface water bodies. Using the resulting GIS layers, the total crop area in vulnerable soils was calculated based on the previously calculated densities and adjustment factors.

To understand the uncertainty associated with the use of specific land cover datasets, an overlay assessment was conducted using alternative data sources. In this approach, the land cover for Western Africa was first extracted from the full global land cover datasets using the ArcGIS “select by mask” geoprocessing tool. Once individual layers were created, these datasets were combined into a single dataset using the ArcGIS “Combine” geoprocessing tool. This new single layer contains both the data source used in this study, and the alternative data source. To determine which cropland areas were common between the layers and which were unique to each layer, a series of queries were executed. The cropland area was calculated and divided by the area from the dataset used in this study to determine the percent spatial overlap.

## Results and discussion

Agricultural land uses account for just over 15% of all land use in the CILSS region. Using the ESA GlobCover, the highest densities of agricultural production areas are found in southern Burkina Faso, Mali, Chad and Senegal. The PUA for acetochlor, which are the combined production areas of corn and cotton, are located chiefly in a band stretching across the CILSS region from the southern regions of Mauritania, Mali, Niger, Chad, across all of Burkina Faso, Senegal, Gambia and Guinea-Bissau (Fig 9). The potential use area of corn and cotton, based on the combination of ESA GlobCover and mapSPAM data, is calculated to be 2,185,987 ha. This represents approximately 0.4% of the total land area or 2.5% of all agricultural areas in the CILSS region. PUAs range widely, from 0 ha in Cabo Verde, to 16,477 ha in Mauritania, to over 800,000 ha (in Mali and Burkina Faso; Table 1). The top three countries in production of these crops are Burkina Faso accounting for 38.8% of the potential uses areas, Mali 37.5%, and Chad 12.8%. When the land areas are expressed in percentages of total agriculture in a CILSS member state, Burkina Faso, Gambia and Mali have the highest percentages of potential uses areas at 4.5%, 4.1% and 3.0% respectively (Table 2). This demonstrates that overall potential use of acetochlor in corn and cotton is limited to a small fraction of the agricultural landscape in the CILSS region.

**Table 1:** Total crop area (ha) in the potential use area, overlapping vulnerable soils, within proximity of surface water or overlapping shallow groundwater within the CILSS region analyzed.

Country	Potential use area			Vulnerable soils			Surface water^a^ assessment			Groundwater^b^ assessment		
	Corn (ha)	Cotton (ha)	Total (ha)	Corn (ha)	Cotton (ha)	Total (ha)	Corn (ha)	Cotton (ha)	Total (ha)	Corn (ha)	Cotton (ha)	Total (ha)
Burkina Faso	362,516	486,016	848,532	13,639	4,750	18,389	1,022	318	1,340	13,434	4,740	18,174
Cabo Verde	0	0	0	-	-	-	-	-	-	-	-	-
Chad	129,434	150,817	280,251	9,204	10,972	20,176	1,045	1,192	2,236	8,778	10,568	19,347
Gambia	25,441	4,527	29,968	96	9	106	0	0	0	0	0	0
Guinea-Bissau	13,540	4,249	17,789	0	0	0	0	0	0	0	0	0
Mali	349,640	469,337	818,977	32,719	53,004	85,723	1,906	3,017	4,923	32,512	52,821	85,334
Mauritania	16,362	115	16,477	9,961	101	10,061	1,331	6	1,337	9,434	99	9,533
Niger	13,167	7,145	20,313	8,455	4,633	13,087	1,837	463	2,300	7,693	4,386	12,079
Senegal	118,097	35,582	153,679	14,037	109	14,146	706	6	712	14,009	109	14,118
**Total of analyzed CILSS countries**	**1,028,198**	**1,157,789**	**2,185,987**	**88,110**	**73,578**	**161,688**	**7,847**	**5,002**	**12,849**	**85,861**	**72,723**	**158,584**

^a^Surface water assessment used a proximity of 200m instead of 20m. The standard 20m (66ft) buffer would have results in no vulnerable areas in proximity to surface water.

^b^Groundwater assessment assumes shallow groundwater is less than 9m deep.

**Table 2:** Percentage potential use area, overlapping vulnerable soils, within proximity of surface water or overlapping shallow groundwater within the CILSS region analyzed.

Country	Potential use urea (PUA) as percentage of agriculture in a CILSS country			PUA as a percentage of vulnerable soils in the country			Surface water^a^ assessment percent crop area on vulnerable soils			Groundwater^b^ assessment percentage of PUA on vulnerable soils		
	Corn (%)	Cotton (%)	Total (%)	Corn (%)	Cotton (%)	Total (%)	Corn (%)	Cotton (%)	Total (%)	Corn (%)	Cotton (%)	Total (%)
Burkina Faso	1.9	2.6	4.5	3.8	1.0	2.2	0.3	0.1	0.2	3.7	1.0	2.1
Cabo Verde	0.0	0.0	0.0	-	-	-	-	-	-	-	-	-
Chad	0.7	0.8	1.6	7.1	7.3	7.2	0.8	0.8	0.8	6.8	7.0	6.9
Gambia	3.5	0.6	4.1	0.4	0.2	0.4	0.0	0.0	0.0	0.0	0.0	0.0
Guinea-Bissau	1.0	0.3	1.3	0.0	0.0	0.0	0.0	0.0	0.0	0.0	0.0	0.0
Mali	1.3	1.7	3.0	9.4	11.3	10.5	0.5	0.6	0.6	9.3	11.3	10.4
Mauritania	0.4	0.0	0.4	60.9	87.8	61.1	8.1	5.6	8.1	57.7	86.2	57.9
Niger	0.4	0.2	0.6	64.2	64.8	64.4	14.0	6.5	11.3	58.4	61.4	59.5
Senegal	0.9	0.3	1.2	11.9	0.3	9.2	0.6	0.0	0.5	11.9	0.3	9.2
**Total of analyzed CILSS countries**	**1.2**	**1.3**	**2.5**	**8.6**	**6.4**	**7.4**	**0.8**	**0.4**	**0.6**	**8.4**	**6.3**	**7.3**

^a^Surface water assessment used a proximity of 200m instead of 20m. The standard 20m buffer would have resulted in no vulnerable areas in proximity to surface water.

^b^Groundwater assessment assumed shallowed groundwater is less then 9m deep

**Fig 10:**
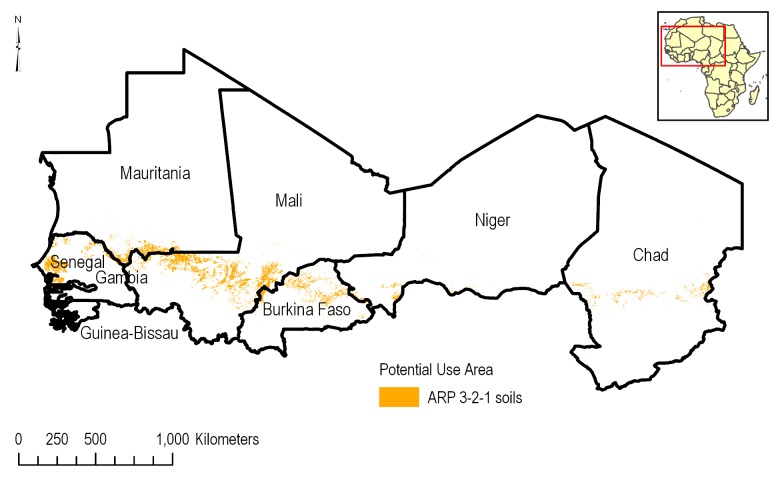
Spatial distribution of the ARP 3-2-1 soils in the PUA.

Soils adhering to the ARP 3-2-1 criteria under agricultural conditions are distributed in a narrow band across the transitional zone from deserts in the north to mixed land use in the south of the CILSS region (Fig 10). These soils are more prominent from western Niger to the west coast in Gambia and Senegal. The total PUA area as a percentage of agricultural area in each CILSS country is shown in Table 2. ARP 3-2-1 soils can be found across Mauritania, Niger, the northern regions of Chad, Burkina Faso, Gambia and Senegal in a narrow band. By percentage, Mauritania and Niger have the highest density of PUA as a percentage of ARP soils at 61.1% and 64.8% respectively. Overall 7.4% of the potential use area has ARP 3-2-1 soils. The Sahara Desert region soils also possesses many soils that fall under the ARP 3-2-1 soils criteria because of the sandy soil texture and extremely low (0%) organic matter content; however, this region does not present a significant growth area for corn/cotton and was thus not included in the use site assessment.

ARP 3-2-1 soil presence in CILSS countries varies widely, accounting for as little as 0.4% of all agricultural soils in Gambia to 64.4% in Niger. Higher percentages of ARP 3-2-1 soils in agricultural areas of Niger indicate that more of the corn and cotton production occurs in soils that are potentially sensitive to leaching in that country (Table 2). Across all CILSS countries in the analysis, slightly more ARP 3-2-1 soils are under corn production (8.4%) versus cotton production (6.3%). Niger and Mauritania have the highest percentages of corn production on ARP 3-2-1 soils at 57.7% and 58.4% respectively whereas Burkina Faso has 3.7%. Most cotton, 86.2%, in Mauritania overlaps shallow groundwater and is grown on ARP 3-2-1 soils.

Shallow groundwater, where the water table is less than 9 m deep, is present in 40.3% of the CILSS agricultural productions areas considered in this study. Shallow groundwater is scattered throughout the entire analyzed region but is mostly concentrated along river beds in the southern portion of the CILSS with extensions into the more arid regions in the north.

**Fig 11:**
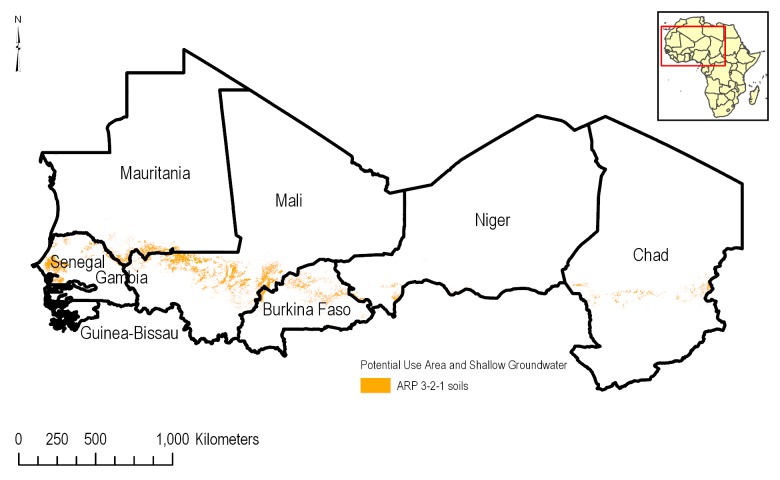
Spatial distribution of the ARP 3-2-1 soils in the PUA overlapping shallow groundwater.

A total of 158,584 ha (7.3%) of the PUA occur where ARP 3-2-1 soils are combined with shallow groundwater (Fig 11). The highest percentages are reported for Mali (10.4%), Mauritania (57.9%) and Niger (59.5%). These percentages, though relatively high, represent only 85,344 ha in Mali, 9,533 ha in Mauritania and 12,079 ha in Niger.

ARP 3-2-1 soils adjacent to surface water bodies ranged from 712 ha (0.5%) in Senegal to 4,923 ha (0.6%) in Mali. For Cabo Verde, Gambia and Guinea-Bissau, no ARP 3-2-1 soils were found to be adjacent to surface water resources. Overall 12,849 ha (0.6%) of all the PUA on corn and cotton are adjacent to surface waters in the CILSS region. This is a small fraction of the corn and cotton agricultural lands. Although a buffer distance of 20 m (66 ft) is recommended by the ARP for rivers and 61 m (200ft) for lakes, due to data resolution concerns, a buffer distance 200m (656 ft) was used to calculate the areas. If a buffer distance of 20 m is applied, no PUA on corn and cotton are adjacent to surface waters in the CILSS region.

## Discussion

This spatial assessment of the PUA demonstrated that few areas in the analyzed CILSS region are vulnerable to acetochlor leaching or runoff, given the ARP 3-2-1 soil criteria. To our knowledge, no other landscape level studies were conducted in western Africa to quantify the fraction of agricultural areas vulnerable to leaching or runoff of a specific pesticide. For example, Jovanovic et al. [15] and Thioune et al. [16] used GIS overlay methods such as SI or DRASTIC to assess groundwater vulnerability across the entire landscape but did not refine it to areas where applications may occur or adapt the method for a product specifically.

As with any GIS assessment, availability of suitable datasets is a requirement. For many countries in the CILSS, no detailed high-resolution local datasets are available. To resolve this deficiency, several global databases were used instead. The use of global datasets for local assessments is not without uncertainty. Spatial resolution and age of the data are of prime concerns. Regarding the coarseness of the cropping data for example, 10 km x 10 km can be considered as a potential drawback as it may over-represent the spatial extent of production. However, it can reasonably be argued that this coarser resolution provided a more conservative approach as more of the landscape in the CILSS region was consequently included in the assessment.

The main reason for using the mapSPAM data was to determine where corn and cotton were likely to be produced within areas classified as agriculture. Using the FAO Agricultural Statistics Database (FAOSTATS) [17] would be a logical choice to determine the corn and cotton production areas in Western Africa. However, the data is not available for cotton and is provided as tabular information at the country level. Redistributing country-level data would result in uncertainty as data would be homogenously distributed over any agricultural area, thus not accounting for variability in production across the CILSS region or even within a country. Using mapSPAM, which relies on FAO data, has the benefit that the spatial-redistribution process is documented and applied the same to both corn and cotton. An assessment shows that 61% of the cotton production overlaps with the corn production at the 10 km x 10 km level. This may indicate that precise field-scale information for a corn-cotton crop rotation system is not readily captured by a large grid such as a 10 km x 10 km pixel.

**Fig 12:**
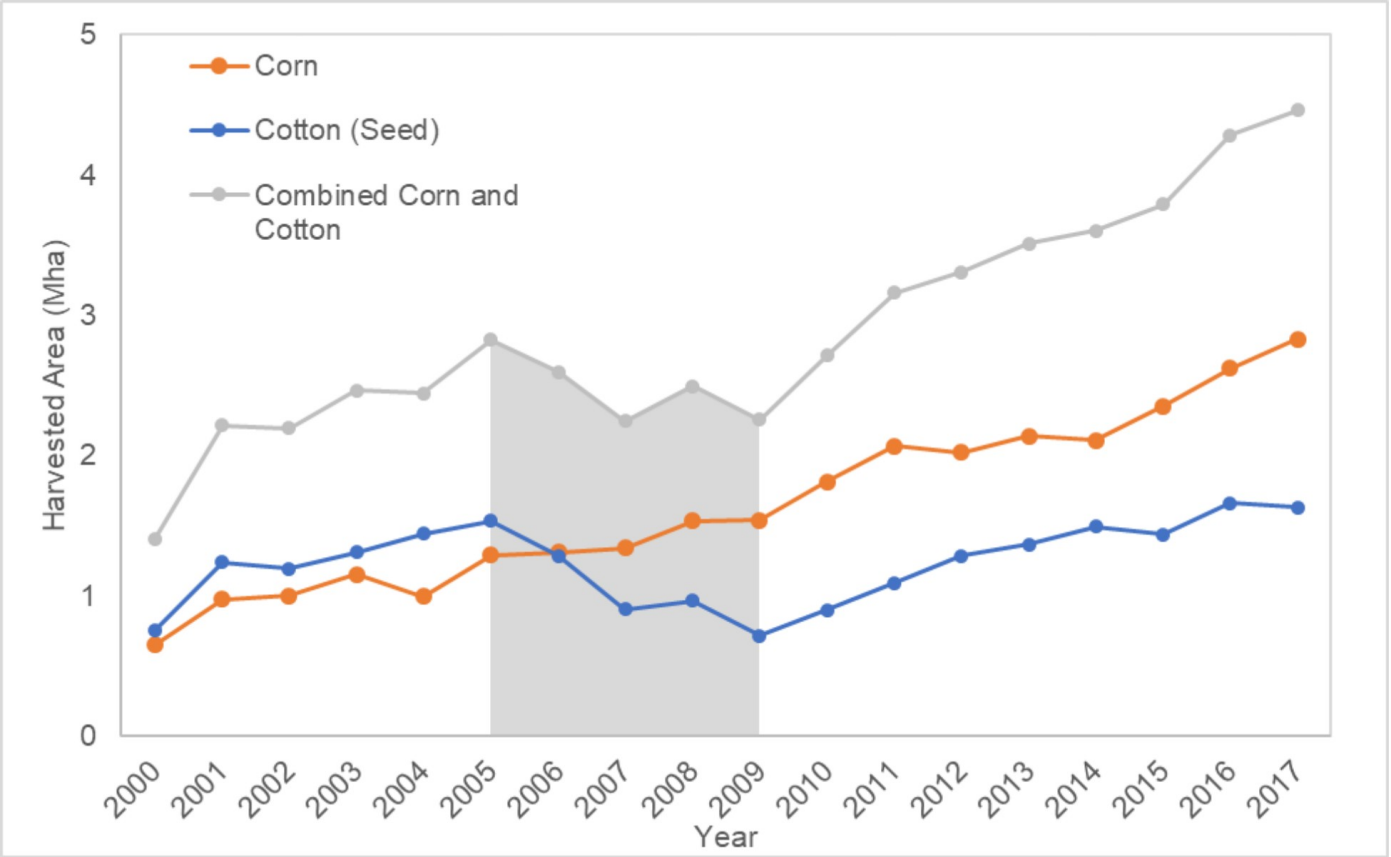
Total corn and cotton production in Western Africa from 2000–2017 based on FAO statistics [17]. The period 2005–2009 is highlighted in grey to emphasize the total production trend in that time period.

FAOSTATS [17] for the combined Western Africa countries (Fig 12) shows that the combined total production of corn and cotton dropped during the period 2005–2009. For the stated period, there was a decrease and increase for cotton and corn, respectively. Consequently, the temporal offset caused by using mapSPAM based on FAOSTAT 2005 instead of 2009, increased the combined total area of cropland under corn or cotton production. Relative to 2005, corn production increased by 19.2% and cotton production decreased by 53%. There was an overall decrease of 20.2% for the combined harvested acres of both crops. It can be concluded that this assessment overestimated the combined total cropland (corn and cotton production) on vulnerable soils, given that the total harvested acres was considered in the evaluation. The breakdown of crop production by individual country is provided in S1 Fig.

The mapSPAM database has been evaluated by several institutions [18,19]. Tan et al. [19], assessed the use of mapSPAM in China for three staple crops, rice, wheat, and maize. Based on this research it was concluded that the map of maize has the highest area accuracy (64%), but accuracy was lower for wheat and rice. On a subnational level mapSPAM did well for the crops in the top 10 producing provinces, but less so for other provinces. The dependency on national or subnational level agricultural statistics was demonstrated by Joglekar et al. [20] who found that SPAM2005 estimates are most dependent on the degree of disaggregation of the underlying national and subnational production statistics. For Nigeria, a low spatial similarity index (SSI) of 0.241 for harvested area was calculated when only national level statistics were used.

An alternative to FAOSTAT and mapSPAM is CELL5M [21,22]. This is a geospatial dataset a 5-min arc spatial interval (roughly 10 km) containing over 750 data layers, including 134 layers focusing on crop production. A potential strength of this dataset is that it includes access to markets to support agricultural development and other factor to account for regions where crops are potentially produced. Technically this could improve crop production estimates. However, the primary underlying datasets for CELL5M are the same datasets as mapSPAM and include FAOSTAT and SPAM itself. No information has been found on the accuracy of the CELL5M dataset.

In Western Africa insufficient information is available at subnational level to generate reliable crop production estimates. Irrespective of the which dataset will be utilized for spatial analysis, much uncertainty remains with respect to crop production and location of agriculture in regions such as the CILSS. Particularly in Western Africa insufficient information was available at subnational level to generate reliable crop production estimates.

Several global soils datasets were considered for use in assessing ARP 3-2-1 soils in the CILSS region. Among these datasets were the Harmonized World Soils Database [23] African Soils Information System (AfSIS; [24]) and SoilGrid [10]. Harmonized World Soils Database (HWSD) was released in 2009 and incorporated state-of-the-art soils databases such as the 1:1,000,000 scale Soil Map of China [25], the Soil Database for Europe [26], and the WISE soil profile database [27]. Resulting was a standardized database for the world having over 16,000 mapping units with robust associated attribute data. Accuracy and reliability of the HWSD is variable and greatly depends on the source data used. North America, Australia, West Africa (excluding Senegal and Gambia) and South Asia are considered less reliable, while most of the areas covered by the Soil and Terrain (SOTER) databases are considered to have the highest reliability (Southern and Eastern Africa, Latin America and the Caribbean, Central and Eastern Europe).

AfSIS [23], was released in 2015 and is best considered as a stop-gap soil information database for Africa. It is based on 18,000 unique soil profiles across the continent gleaned from the African Soil Profile Database [28]. Spatial predictions for selected soil properties relevant to agriculture were generated. Key outputs for seven different depths included organic carbon, particle size distribution, and bulk density. AfSIS was subsequently superseded by SoilGrid in 2017.

SoilGrid [10] represents the latest and highest-resolution soils dataset for the world. A first release in 2014 [24] was considered a proof-of-concept at 1 km resolution and demonstrated that “global compilations of soil profiles can be used in an automated framework to produce complete and consistent spatial predictions of soil properties and classes”. A major concern was the overestimation of low values for many of the soil properties. The release of SoilGrid in 2017 at 250 m resolution addressed many of the concerns of the proof-of-concept database. In the 2017 version, staff used a complex combination of machine learning, non-linear interpolation techniques, and an ensemble prediction framework to improve the soil property estimates at the landscape level. Predictions were based on over 150,000 data points across the globe, with 18,000 of these points occurring in Africa. These were based on the African Soil Profile database [28]. Of these points, 2,786 fall within the CILSS region analyzed. Notably, the Sahara region has no profile data in the database. This supports the decision to exclude Saharan regions of the CILSS from analysis, as any predictions in the regions would have considerable uncertainty and represent faulty associations with default values for texture and organic carbon in the northern region of the CILSS.

Issues resulting from the use of global datasets may also manifest in representations of surface water, since small bodies of water such as ponds and agricultural streams may not be captured at the native resolution. Resulting from this omission is that not all PUA overlapping ARP 3-2-1 soils were included in the assessment. Therefore, potentially vulnerable areas could still exist outside of the parameters of this assessment but would require very high-resolution local data to accurately identify.

### Challenges with land cover data

The rapid developments in remote sensing techniques, changes in land use cover, and temporal offsets were factors considered during the dataset review and selection for this study. Several global land cover (GLC) products were considered for use in this assessment including AFRICOVER [29], University of Maryland Global Land Cover Facility (UMD GLCF) [30], GlobCover 2005 [31] and GlobCover 2009 [8,32]. Newer datasets such as the GlobeLand30m [33] and LC-CCI 2010 [34] were also reviewed. A brief overview and discussion on the accuracy and quality of several GLC datasets is provided below.

AFRICOVER [29] was released in 2000 at a spatial resolution of 30m based on the LANDSAT TM images (bands 4,3,2) acquired for the periods 1982 to 2000. The land cover classes have been developed using the FAO/UNEP international standard Land Cover Classification System (LCCS). Since its initial release, AFRICOVER has been updated for several African countries, except for the Western Sahel region. The UMD GLCF consists of four datasets at a 1 km resolution and represents the following four years (1975, 1990, 2000 and 2005). The data are based on the AVHRR Pathfinder 1 km sensor and a single class is used to represent cropland. The 2000 dataset shows very little cropland in Western Africa and was therefore rejected. No assessment of the 2005 dataset was conducted, because this dataset could not be obtained.

The European Space Agency (ESA) GlobCover project aim was to develop a service capable of delivering global land cover maps using as input observations from the MERIS sensor on board the Environmental Satellite (ENVISAT). ESA made available two data products covering the periods December 2004 to June 2006 and January to December 2009, referred to as the 2005 and 2009 datasets, respectively. Both products use the 22 FAO Land Cover Classification System (LCCS) scheme, four of which cover agricultural production. The accuracy of the 2005 and 2009 datasets are 74% and 68%, respectively [8,31,32].

As part of the ESA Climate Change Initiative (CCI), ESA’s Land Cover (LC) project delivered three consecutive GLC maps at a 300 m resolution using the MERIS data for the time series 1998–2002 (dataset, 2000), 2003–2007 (dataset, 2005) and 2008–2012 (dataset, 2010) [34]. The generated maps have a classification scheme with 22 classes for the FAO land cover classification and were specifically targeted to meet the requirements of climate modelers. The overall thematic accuracy of the LC-CCI 2010 map is 74%.

Accuracy and quality of cropland data derived from satellite imagery has been assessed by many researchers. In several of these studies, Africa [35,36,37] or Western Africa [38,39] was the focus. It was reported that newer datasets such as FAO-GLCshare and Globeland30 were adequate to properly classify cropland compared to older dataset such as GlobCover 2009 and CCI Land Cover 2010. Discrepancies in cropland between the land cover datasets has been well-documented [36,37,38]. Researchers concluded that within a country the quality of the dataset can vary greatly and that no single dataset covers cropland, specifically in Africa, with a high degree of resolution or accuracy. Fritz et al. [35,40] recognized that large discrepancies between current continental and global land cover maps exist both in terms of overall area and spatial distribution particularly for Africa.

To resolve the uncertainty in GLC datasets, mapSPAM [9] developed an approach that involved combining five land cover products (GLC‐2000, MODIS Land Cover, GlobCover, MODIS Crop Likelihood and AFRICOVER) into a single synergy map with an estimated accuracy of 83%. By comparison GlobCover 2005 has 74% accuracy, GlobCover 2009 has 68% accuracy and the CCI-LC 2010 product has 74% accuracy and GlobeLand30m has 80% accuracy. The newer GlobeLand30m [33] and LC-CCI 2010 [34] datasets fell outside the temporal envelope to be used in conjunction with mapSPAM. It is worth noting that mapSPAM 2005 uses a variety of global and regional land cover datasets publicly available for various years and includes GlobCover 2005 and not CCI-LC. In comparison to GlobCover 2005, GlobCover 2009 has greater spatial extent that was desirable to capture variability in soils, albeit introducing additional uncertainty with respect to the location of crops. Future work could include temporally aligning the mapSPAM data (2010 version-release Dec 2018) with a suitable medium resolution land cover layer such as 2010 CCI LC or other suitable datasets to limited to temporal offset between the dataset.

There is much disagreement in which areas are defined as cropland when comparing the different datasets. Areas of full disagreement are more abundant in Africa (30%) [36]. A general low correspondence between the dataset in Western Africa especially in the non-desert areas has been observed [38]. Samassee et al. [39] determined that GFSAD30 and GlobeLand30 present better accuracy in identifying crop areas. They have, in the Sahel, an average cropland class accuracy of 69% and 64% for GlobeLand30 and GFSAD30, respectively although both tend to underestimate crop areas. Assessment of cropland by Yanbing et al. [37] show that GlobeLand30 has the best statistical fit compared to observed data in China, followed by MODIS Collection 5 and Unified Cropland, GlobCover and CCI Land Cover have the lower accuracies.

**Fig 13:**
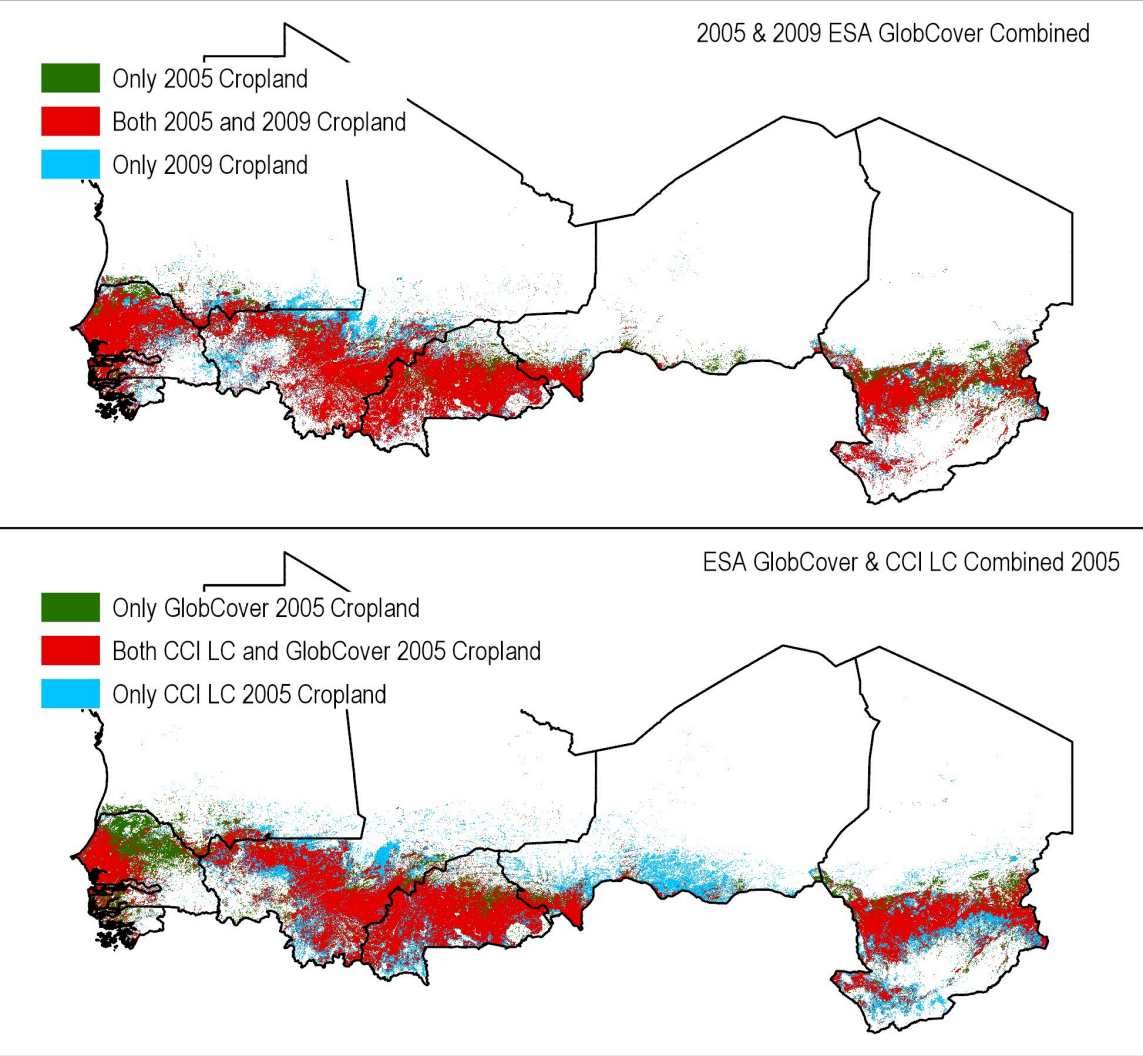
Differences in spatial coverage for agricultural lands for ESA 2005 [31] and 2009 GlobCover [8] (top), GlobCover 2005 and ESA CCI LC 2005 [34] (bottom).

An assessment of cropland distribution between several of the considered land cover datasets demonstrate these differences. For example, when Globcover 2005, 2009 and CCI-LC 2005 are compared (Fig 13), much discrepancy is observed. GlobCover 2005 and 2009 have 85% overlap and GlobCover 2009 has 6% more cropland mapped, thus providing a greater spatial extent. When GlobCover 2005 is compared to CCI-LC 2005, 78% overlap between cropland of both datasets exists using the 2005 GLC as the basis. Table 3 details the difference in cropland area between the various datasets. It is shown that cropland areas in Bukina Faso, Niger and Chad are underestimated by GlobCover compared to CCI-LC. However, Globcover has more cropland in Senegal and Mali. Consequently, our assessment could potentially overestimate the areas of vulnerable soils in northeastern Mali and Senegal, but underestimate in Burkina Faso, Niger and Chad when compared with CCI-LC dataset.

**Table 3:** Differences in impacted cropland with reported corn and cotton production based on distinct versions of global land cover datasets. Percentages are based on the combined datasets area and not on an individual GlobCover dataset.

	Percent Cropland Overlap for GlobCover (2005 and 2009)*			Percent Cropland Overlap for GlobCover (2009) and CCI LC (2009)*		
Country	Reported as cropland in 2005 only	Merged 2005 & 2009	Reported as cropland in 2009 Only	Reported as cropland in CCI LC Only	Merged CCI LC & GlobCover	Reported as cropland in GlobCover Only
**Burkina Faso**	8.1%	84.0%	7.9%	16.3%	75.5%	8.2%
**Chad**	21.7%	64.5%	13.8%	35.6%	54.0%	10.5%
**Gambia**	5.0%	85.0%	10.0%	13.2%	72.3%	14.5%
**Guinea-Bissau**	10.1%	58.6%	31.4%	14.9%	38.4%	46.7%
**Mali**	5.6%	70.2%	24.2%	20.8%	61.1%	18.1%
**Mauritania**	19.2%	38.9%	41.9%	20.4%	32.1%	47.5%
**Niger**	27.6%	60.0%	12.5%	68.1%	24.7%	7.2%
**Senegal**	8.6%	76.1%	15.2%	5.2%	44.6%	50.3%

*Percentages are based on the combined datasets area and not on an individual GlobCover dataset

It is evident from this analysis that the results in this manuscript are dependent on the accuracy of the input geospatial datasets. With advances in technology, new and improved datasets will become available. While it is unexpected that overall conclusions of this study to change significantly, it would be useful to generate refined estimates for vulnerable areas in Western Africa as new and improved input datasets become available. Although this analysis will be improved as better data becomes available, growers will still need to evaluate the potential for groundwater or surface water contamination for specific cropland fields.

Although vulnerability studies using widely available data have become more common, only few emphasized the assessment of agricultural chemical use and water resources within western Africa, a prevalent and productive agricultural region. The Sahel region has been relatively poorly studied, although other regions of Africa have been focused on during the last 20 years (Table 4). When little or no data is available to conduct a traditional groundwater and surface water assessments, Geographic Information Systems (GIS) based approaches such as DRASTIC, SI, and SINTACS can provide a suitable substitution given the availability of coarser scale geospatial data. DRASTIC, developed in 1987 by the US Geological Survey (USGS; [41]) was designed for the US EPA to assess aquifer vulnerability. The model considers a contaminant introduced at the surface, which moves downwards due to recharge at a rate equal to water movement. The vulnerability of the aquifer is assessed by examining intrinsic properties of the aquifer and vadose zone, the water table depth, topography, and recharge. Each of the model’s inputs are weighted based on local expertise and setting. DRASTIC has been applied in Africa by Ahmed [42] to conduct a vulnerability assessment of the Quaternary aquifer at Sohag, Egypt. Sustainability Index (SI) is a modification of DRASTIC which includes a factor to account for land use cover [16]. Another improved DRASTIC model was developed to assess contaminants under specific South African environmental conditions [15]. Likewise, a more advanced GIS-based approach using weighted-overlay analysis using the SINTACS method was implemented by Jarray et al. [43] to assess vulnerability of an aquifer in Southern Tunisia. Unlike DRASTIC, the SINTACS method allows the use, at the same time and in different cells, of weighting factors that account for specific environmental conditions. Combined with crop maps, this information can be used to assess the fraction of the PUA vulnerable to pesticides.

**Table 4:** Overview of conducted spatial and water vulnerability studies in Africa.

Author	Year	Model	Variable of Interest	Study Area
Robins et al. [44]	2007	DRASTIC	Aquifer & groundwater vulnerability	Africa
Saayman et al. [45]	2007	AQUISOIL	Aquifer vulnerability	South Africa
		DRASTIC		
		EUZIT		
		Ugif		
		Attenuation Factor		
		Leaching Potential		
		Index		
		Runoff Index		
Ahmed [42]	2009	DRASTIC	Aquifer vulnerability	Egypt
Jovanovic et al. [15]	2006	DRASTIC	Groundwater vulnerability	South Africa
Musekiwa and Majola [46]	2011	DRASTIC	Groundwater vulnerability	South Africa
Mongwe and Fey [47]	2004	DRASTIC	Groundwater vulnerability	South Africa
		SEEPAGE		
Gad et al. [48]	2015	GOD	Groundwater vulnerability	East Delta, Egypt
		PRAST		
		DRASTIC		
Jarray et al. [43]	2016	SINTACS	Groundwater vulnerability	Tunisia
Ouedraogo [49]	2017	DRASTIC	Nitrates	Africa
Thiuone et al. [16]	2017	SINTACS	Nitrates	Senegal
Ouedraogo and Vanclooster [50]	2016	Statistical exploration	Nitrates	Africa
Kawo and Karuppannan [51]	2018	Statistical exploration	Water quality	Ethiopia

Using basic soil criteria, such as the ARP has done, may appear to be a simplistic approach when compared to spatial index methods such as DRASTIC. However, the ARP approach has the advantage that fewer input data are needed and these data represent the key driving factors for acetochlor movement in the environment. It is a balanced screening level approach that is both simple and robust. Most currently available soil datasets have the required textural and organic matter information to allow for the development of models from these criteria.

## Conclusions

A spatial assessment was conducted to determine the fraction of the landscape that represents areas that simultaneously are in proximity to surface or groundwater, adhere to the ARP 3-2-1 soil criteria, and are currently planted with corn or cotton. The combined corn and cotton growing areas, referred to as the Potential Use Areas, or PUA, cover 2,185,987 ha in the CILSS region (excluding Benin, Ivory Coast, Guinea, and Togo). This represents 2.5% of the agricultural landscape. The PUA were assessed with acetochlor in mind and the ARP 3-2-1 soil criteria were used to determine presence of the vulnerable soils. It was revealed that 7.4% of the CILSS region agricultural areas adhere to said criteria. Of the PUA, 7.3% overlap shallow groundwater. The adjacency surface water analysis showed that 0.6% of the potential use area is on ARP 3-2-1 soils. If a buffer distance of 20 m is applied, no PUA on corn and cotton are adjacent to surface waters in the CILSS region. Even using an extremely conservative approach, there are few acetochlor use areas within the CILSS region that present a potential risk for contamination of either surface or groundwater.

Relying on global datasets such as SoilGrid, mapSPAM, and ESA GlobCover in lieu of regional high-resolution data sources, it was demonstrated that these coarser datasets can produce spatial assessments that provide valuable insights across a larger landscape. Overlap between selected land cover datasets in the Sahel region varies and ranges from 24.7% to 75.5% based on a merged 2009 GlobCover and CCI LC datasets. In comparison with the merged 2005 and 2009 GlobCover dataset, the cropland overlaps range from 38.9% to 85.0%. This demonstrates that the choice of land cover dataset can have a significant impact on a spatial assessment.

The results of this study suggest potential local driving factors for use and where best management practices for acetochlor, and most of which are generally applicable to all herbicides, can be applied in a poorly studied region. The easiest of these best management practices to implement, and the most effective for protecting potential sources of drinking water from contamination, is to apply a standard application set-back from all groundwater wells and all surface water sources. Irrespective of any local conditions, farmers should follow recommended best management practices [2] and use products containing acetochlor only according to the approved label.

## Supporting information

S1 Fig Corn and cotton production in Western Africa countries. This figure shows the crop production trend for corn and cotton for individual Western Africa countries for the period 2000–2017. The period 2005–2009 shows an increase in corn but a decrease in cotton (seed) production.
(TIF)
