# Supplementary figures and images for: Assessment of acetochlor use areas in the sahel region of Western Africa using geospatial methods
Source: PLoS One. 2020 May 1;15(5):e0230990. doi: 10.1371/journal.pone.0230990 (PMC7194437; doi:10.1371/journal.pone.0230990)

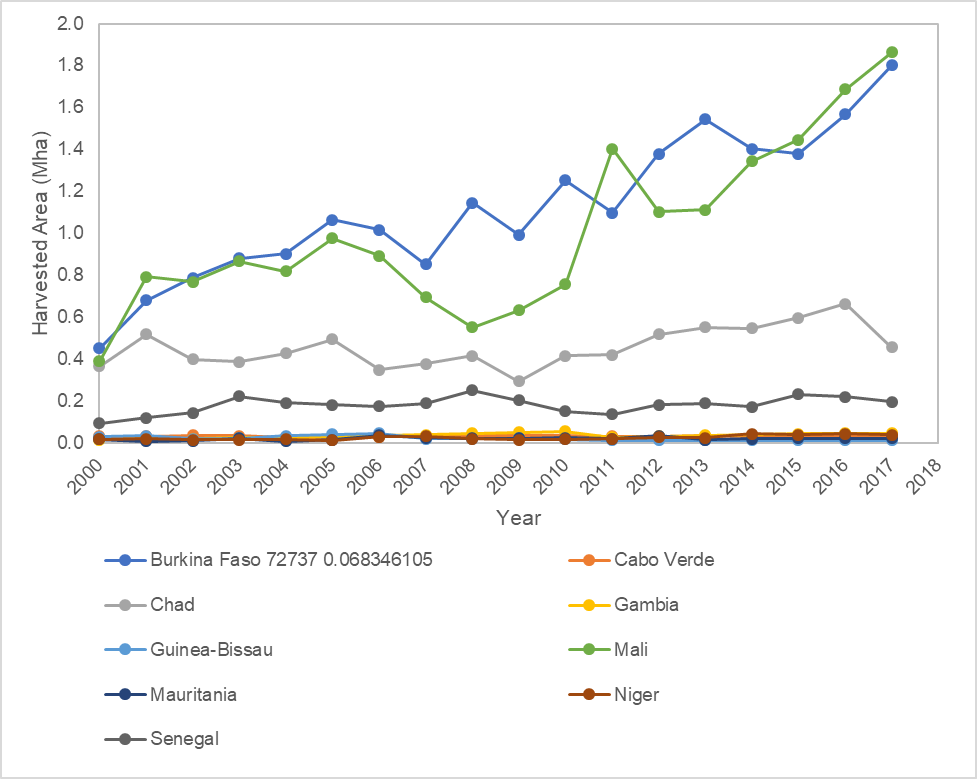

Supplement: S1 Fig — This figure shows the crop production trend for corn and cotton for individual Western Africa countries for the period 2000–2017. The period 2005–2009 shows an increase in corn but a decrease in cotton (seed) production. (TIF) [file pone.0230990.s001.tif]
